# Preclinical evaluation of Gd-DTPA and gadomelitol as contrast agents in DCE-MRI of cervical carcinoma interstitial fluid pressure

**DOI:** 10.1186/1471-2407-12-544

**Published:** 2012-11-22

**Authors:** Tord Hompland, Christine Ellingsen, Einar K Rofstad

**Affiliations:** 1 Group of Radiation Biology and Tumor Physiology, Department of Radiation Biology, Institute for Cancer Research, Oslo University Hospital, Nydalen, Box 4953, Oslo, N-0424, Norway

**Keywords:** Cervical carcinoma xenografts, DCE-MRI, Gadomelitol, Gd-DTPA, Interstitial fluid pressure

## Abstract

**Background:**

High interstitial fluid pressure (IFP) in the primary tumor is associated with poor disease-free survival in locally advanced cervical carcinoma. A noninvasive assay is needed to identify cervical cancer patients with highly elevated tumor IFP because these patients may benefit from particularly aggressive treatment. It has been suggested that dynamic contrast-enhanced magnetic resonance imaging (DCE-MRI) with gadolinium diethylene-triamine penta-acetic acid (Gd-DTPA) as contrast agent may provide useful information on the IFP of cervical carcinomas. In this preclinical study, we investigated whether DCE-MRI with contrast agents with higher molecular weights (MW) than Gd-DTPA would be superior to Gd-DTPA-based DCE-MRI.

**Methods:**

CK-160 human cervical carcinoma xenografts were subjected to DCE-MRI with Gd-DTPA (MW of 0.55 kDa) or gadomelitol (MW of 6.5 kDa) as contrast agent before tumor IFP was measured invasively with a Millar SPC 320 catheter. The DCE-MRI was carried out at a spatial resolution of 0.23 × 0.23 × 2.0 mm^3^ and a time resolution of 14 s by using a 1.5-T whole-body scanner and a slotted tube resonator transceiver coil constructed for mice. Parametric images were derived from the DCE-MRI recordings by using the Tofts iso-directional transport model and the Patlak uni-directional transport model.

**Results:**

When gadomelitol was used as contrast agent, significant positive correlations were found between the parameters of both pharmacokinetic models and tumor IFP. On the other hand, significant correlations between DCE-MRI-derived parameters and IFP could not be detected with Gd-DTPA as contrast agent.

**Conclusion:**

Gadomelitol is a superior contrast agent to Gd-DTPA in DCE-MRI of the IFP of CK-160 cervical carcinoma xenografts. Clinical studies attempting to develop DCE-MRI-based assays of the IFP of cervical carcinomas should involve contrast agents with higher MW than Gd-DTPA.

## Background

Clinical investigations have shown that the interstitial fluid pressure (IFP) is elevated in many tumor types, including lymphoma, melanoma, breast carcinoma, head and neck carcinoma, and cervical carcinoma [1,2]. In squamous cell carcinoma of the uterine cervix, for example, IFP values up to ~50 mmHg have been measured in untreated tumors, whereas most normal tissues show IFP values ranging from −3 to +3 mmHg [3-5]. The mechanisms leading to interstitial hypertension in malignant tissues have been studied extensively in experimental tumors [1]. These studies have shown that elevated IFP is a consequence of severe microvascular, lymphatic, and interstitial abnormalities. Tumors develop interstitial hypertension because they show high resistance to blood flow, low resistance to transcapillary fluid flow, and impaired lymphatic drainage [6]. The microvascular hydrostatic pressure is the principal driving force for the elevated IFP of malignant tissues [7]. Fluid is forced from the microvasculature into the interstitium where it accumulates, distends the extracellular matrix, and causes interstitial hypertension. Differences in IFP among tumors result primarily from differences in resistance to blood flow caused by differences in the architecture of the microvascular network and from differences in transcapillary fluid flow caused by differences in the permeability of the vessel walls [1,6].

A large prospective study of the association between tumor IFP and outcome of treatment has been carried out in patients with locally advanced cervical carcinoma at Princess Margaret Hospital in Toronto [8,9]. The patients were given radiation therapy without chemotherapy, and IFP and oxygen tension were measured in the primary tumor prior to treatment. The study showed that high IFP was associated with poor disease-free survival independent of conventional prognostic factors, such as tumor size, stage, and lymph node status. Moreover, patients with tumors with high IFP had an increased probability of developing recurrences both locally within the irradiated pelvic region and at distant nonirradiated sites. The independent prognostic effect of IFP for recurrence and survival was strong, whereas the independent prognostic effect of tumor hypoxia was of borderline significance and was limited to patients without nodal metastases [9]. The main findings reported by the Toronto group have been confirmed in a smaller prospective study of cervical carcinoma patients treated with radiation therapy at Chungnam National University Hospital in Daejeon [10]. Taken together, these studies suggest that cervical carcinoma patients with highly elevated tumor IFP may benefit from particularly aggressive treatment.

Tumor IFP was measured with the wick-in-needle technique in these studies [8-10]. This is a highly invasive technique that requires insertion of a fluid-filled 0.5 − 1.0-mm-thick steel needle into the tumor tissue [7]. Multiple measurements with the wick-in-needle technique may lead to erronous IFP readings because of tissue damage and interstitial fluid leakage from the needle insertion sites and, consequently, a noninvasive assay for assessing IFP in cervical carcinoma is highly warranted [1,11]. The possibility that dynamic contrast-enhanced magnetic resonance imaging (DCE-MRI) with gadolinium diethylene-triamine penta-acetic acid (Gd-DTPA) as contrast agent may provide information on the IFP of cervical carcinomas has been investigated by Haider et al. [12]. Thirty-two untreated patients were subjected to DCE-MRI, and significant correlations were found between DCE-MRI-derived parameters and tumor IFP. However, the correlations were too weak to be clinically useful, perhaps because the DCE-MRI was not optimized with the purpose of measuring IFP.

DCE-MRI is an attractive strategy for developing a noninvasive assay of the IFP of tumors because the uptake of MR contrast agents in malignant tissues is influenced significantly by some of the microvascular parameters that are decisive for the magnitude of the IFP (i.e., tumor blood perfusion and vessel wall permeability). The molecular weight of a contrast agent decides whether the uptake is determined primarily by the blood perfusion or primarily by the vessel wall permeability. The uptake of low-molecular-weight contrast agents like Gd-DTPA is governed by the blood perfusion, and with increasing molecular weight, the uptake becomes increasingly more dependent on vessel wall permeability [13,14]. Because the IFP of cervical carcinomas may be influenced significantly by the permeability of the vessel walls [3,6], Gd-DTPA may not be the optimal contrast agent for assessing IFP in cervical cancer, a possibility that was investigated in the present preclinical study. We hypothesized that DCE-MRI with contrast agents with higher molecular weights than Gd-DTPA would provide better measures of tumor IFP than Gd-DTPA-based DCE-MRI. To test this hypothesis, human cervical carcinoma xenografts were subjected to DCE-MRI with Gd-DTPA or gadomelitol as contrast agent before tumor IFP was measured invasively. Gadomelitol is an intermediate-sized contrast agent that shows significant uptake in malignant tissues [15].

## Methods

### Tumor models

CK-160 human cervical carcinoma xenografts growing in adult female BALB/c *nu*/*nu* mice were used as tumor models [16]. Tumors were initiated from cells cultured in RPMI-1640 (25 mmol/L HEPES and l-glutamine) medium supplemented with 13% bovine calf serum, 250 mg/L penicillin, and 50 mg/L streptomycin. Approximately 5.0 × 10^5^ cells in 10 μL of Hanks’ balanced salt solution were inoculated in the gastrocnemius muscle. Tumors with volumes of 100–800 mm^3^ were included in the study. DCE-MRI and IFP measurements were carried out with mice anesthetized with fentanyl citrate (0.63 mg/kg), fluanisone (20 mg/kg), and midazolam (10 mg/kg). Animal care and experimental procedures were in accordance with the Interdisciplinary Principles and Guidelines for the Use of Animals in Research, Marketing, and Education (New York Academy of Sciences, New York, NY).

### Contrast agents

Two contrast agents were evaluated: Gd-DTPA (Magnevist®; Schering, Berlin, Germany) with a molecular weight of 0.55 kDa and gadomelitol (Vistarem®; Guerbet, Roissy, France) with a molecular weight of 6.5 kDa. The contrast agents were diluted in 0.9% saline to a final concentration of 60 mM (Gd-DTPA) or 7.0 mM (gadomelitol) and were administered in the tail vein in a bolus dose of 5.0 mL/kg. The administration was carried out after the mice had been positioned in the MR scanner by using a 24 G neoflon connected to a syringe by a polyethylene tubing.

### DCE-MRI

DCE-MRI was carried out as described earlier [17]. Briefly, *T*_1_-weighted images (TR = 200 ms, TE = 3.5 ms, and α_*T1*_ = 80°) were recorded at a spatial resolution of 0.23 × 0.23 × 2.0 mm^3^ and a time resolution of 14 s by using a 1.5-T whole-body scanner (Signa; General Electric, Milwaukee, WI) and a slotted tube resonator transceiver coil constructed for mice. The coil was insulated with styrofoam to prevent excessive heat loss from the mice. The body core temperature of the mice was kept at 37 − 38°C during imaging by using a thermostatically regulated heating pad. Two calibration tubes, one with 0.5 mM (Gd-DTPA) or 0.06 mM (gadomelitol) of contrast agent in 0.9% saline and the other with 0.9% saline only, were placed adjacent to the mice in the coil. The tumors were imaged axially in a single section through the center by using an image matrix of 256 × 128, a field of view of 6 × 3 cm^2^, and one excitation. Two proton density images (TR = 900 ms, TE = 3.5 ms, and α_*PD*_ = 20°) and two *T*_1_-weighted images were acquired before the contrast was administered, and *T*_1_-weighted images were recorded for 15 min after the contrast administration. Contrast agent concentrations were calculated from signal intensities by using the method of Hittmair et al. [18]. The DCE-MRI series were analyzed on a voxel-by-voxel basis by using the iso-directional transport model of Tofts et al. [14] and the uni-directional transport model of Patlak et al. [19].

According to the Tofts model,

$$C_{t}\left(T\right)=\frac{K^{\mathrm{trans}}}{\left(1-Hct\right)}\int_{0}^{T}C_{a}\left(t\right)\cdot e^{\left(-K^{\mathrm{trans}}\cdot \left(T-t\right)/v_{e}\right)}dt+V_{b}^{\mathrm{Tofts}}\cdot C_{a}\left(T\right)$$

where *C*_t_(*T*) is the concentration of contrast agent in the tissue at time *T*, *C*_a_(*T*) is the arterial input function, *Hct* is the hematocrit, *K*^trans^ is the volume transfer constant of the contrast agent, *v*_e_ is the fractional distribution volume of the contrast agent in the tissue, and *V*_b_^Tofts^ is the fractional blood volume of the tissue [14]. Parametric images of *K*^trans^*v*_e_, and *V*_b_^Tofts^ were determined from the best curve fits to plots of *C*_t_*versus T*.

The uni-directional transport model of Patlak et al. [19] is based on the assumption that the transfer of contrast agent from blood to tissue is irreversible and obeys first-order kinetics. According to this model,

$$\frac{C_{t}\left(T\right)}{C_{a}\left(T\right)}=\frac{K_{i}}{\left(1-Hct\right)}\cdot \frac{\int_{0}^{T}C_{a}\left(t\right)dt}{C_{a}\left(T\right)}+V_{b}^{\mathrm{Patlak}}$$

where *C*_t_(*T*) is the tissue concentration of contrast agent at time *T*, *C*_a_(*T*) is the concentration of contrast agent in the blood at time *T*, *Hct* is the hematocrit, *K*_i_ is the influx constant of the contrast agent from the blood to the tissue, and *V*_b_^Patlak^ is the fractional blood volume of the tissue [19]. Plots of *C*_t_(*T*)/*C*_a_(*T*) *versus* ∫*C*_a_(*t*)dt/*C*_a_(*T*) are linear when the assumptions of the model are fulfilled. Parametric images of *K*_i_ and *V*_b_^Patlak^ were determined by fitting linear curves to the data acquired 1–6 min after the contrast administration.

By analyzing blood samples [20,21], the arterial input functions were found to be double exponential functions

$$C_{a}\left(T\right)=A\cdot e^{-B\cdot T}+C\cdot e^{-D\cdot T}$$

with constants: *A* = 2.55 mM, *B* = 0.080 s^−1^, *C* = 1.20 mM, and *D* = 0.0010 s^−1^ (Gd-DTPA) and *A* = 0.086 mM, *B* = 0.043 s^−1^, *C* = 0.363 mM, and *D* = 0.0025 s^−1^ (gadomelitol).

### Interstitial fluid pressure

IFP was measured in the center of the tumors with a Millar SPC 320 catheter equipped with a 2 F Mikro-Tip transducer (Millar Instruments, Houston, TX) [22]. The catheter was connected to a computer via a Millar TC-510 control unit and a model 13-66150-50 preamplifier (Gould Instruments, Cleveland, OH). Data acquisition was carried out by using LabVIEW software (National Instruments, Austin, TX).

### Statistical analysis

Curves were fitted to data by regression analysis. The Pearson product moment correlation test was used to search for correlations between parameters. Probability values (*P*) and correlation coefficients (*R*^2^) were calculated by using SigmaStat software (SPSS Science, Chicago, IL). A significance criterion of *P* < 0.05 was used.

## Results

**Figure 1:**
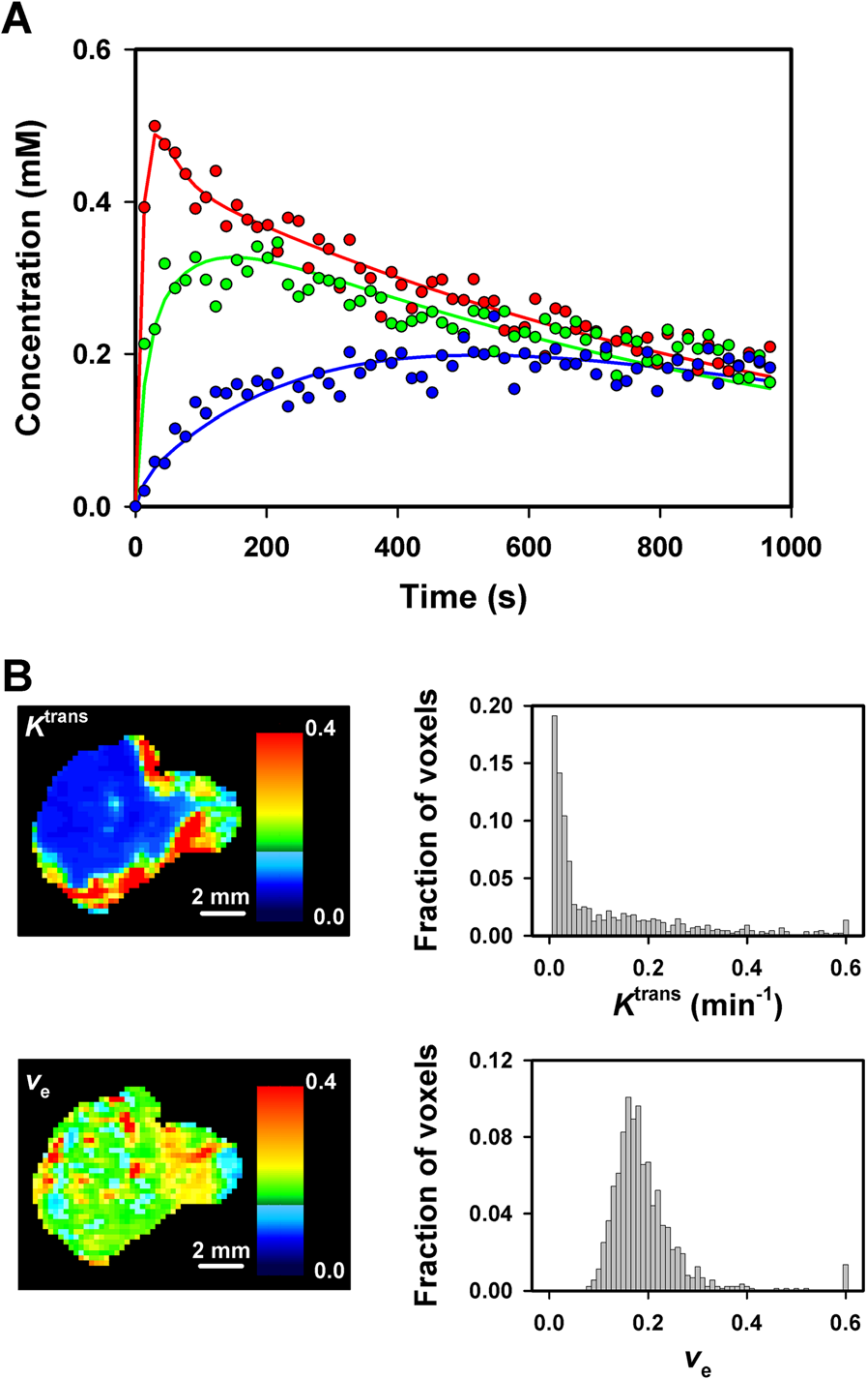
**DCE-MRI data for CK-160 cervical carcinoma xenografts imaged with Gd-DTPA as contrast agent.** (**A**) Gd-DTPA concentration *versus* time for three representative single voxels of a tumor. The curves were fitted to the data by using the Tofts pharmacokinetic model. (**B**) The parametric images of *K*^trans^ and *v*_e_ and the corresponding *K*^trans^ and *v*_e_ frequency distributions of a representative tumor.

DCE-MRI with Gd-DTPA as contrast agent was carried out on eighteen tumors. The plots of *C*_t_(*T*)/*C*_a_(*T*) *versus* ∫*C*_a_(*t*)dt/*C*_a_(*T*) were not linear, most likely because the assumptions of the uni-directional transport model of Patlak were not fulfilled and, consequently, reliable images of *K*_i_ and *V*_b_^Patlak^ could not be established for Gd-DTPA. In contrast, the Tofts model gave good curve fits to the plots of *C*_t_*versus T*, but the uncertainty in the calculations of *V*_b_^Tofts^ were too large that reliable values for this parameter could be obtained, probably because the temporal resolution of the DCE-MRI was not sufficiently high. The curve fitting with the Tofts model was therefore carried out by ignoring the signal from the tumor blood plasma (i.e., *V*_b_^Tofts^ was set to zero). The quality of the curve fitting is illustrated in Figure 1A, which refers to three representative single voxels differing in the rates of uptake and wash-out of Gd-DTPA. Parametric images of *K*^trans^ and *v*_e_ and the corresponding *K*^trans^ and *v*_e_ frequency distributions of a representative tumor are presented in Figure 1B. In general, the tumors were highly heterogeneous in *K*^trans^ with the highest values in the periphery and the lowest values in the center. The intratumor heterogeneity in *v*_e_ was also substantial, but did not follow a fixed pattern (i.e., low and high values were seen in the center as well as in the periphery of the tumors).

**Figure 2:**
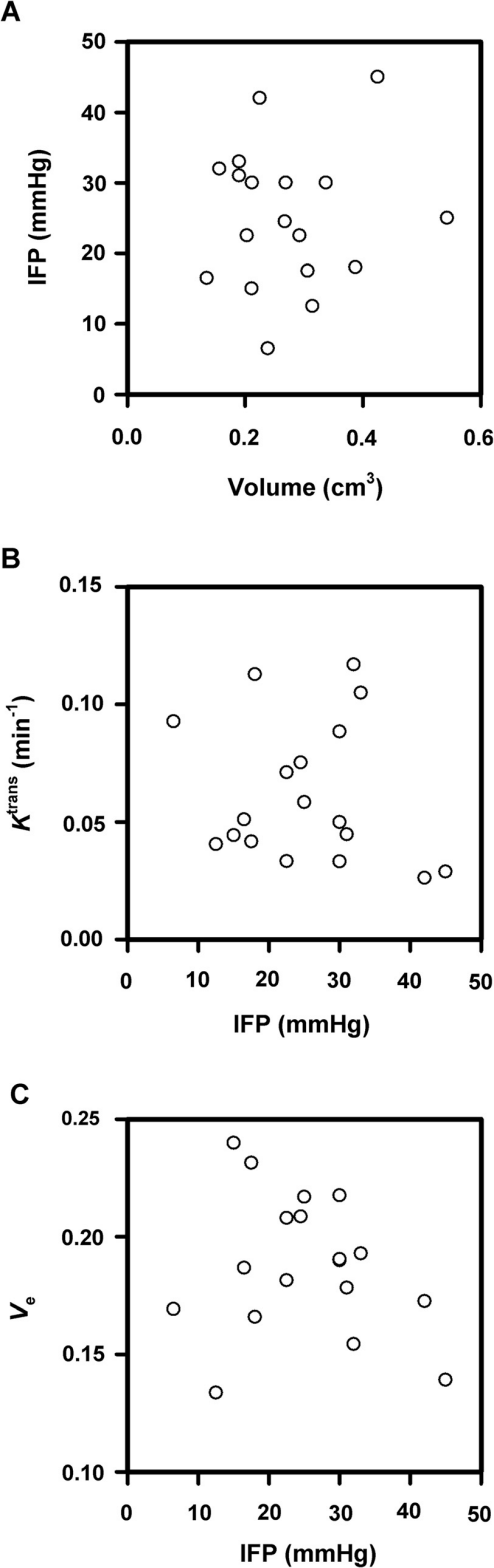
**DCE-MRI and IFP data for CK-160 cervical carcinoma xenografts imaged with Gd-DTPA as contrast agent.** (**A**) IFP *versus* tumor volume. (**B**) Median *K*^trans^*versus* IFP. (**C**) Median *v*_e_*versus* IFP. The points represent single tumors.

IFP was measured immediately after the DCE-MRI and was found to vary among the tumors from 6.5 to 45 mmHg. There was no correlation between IFP and tumor volume (Figure 2A). Moreover, correlations between IFP and *K*^trans^ or *v*_e_ were not found, as illustrated in Figure 2, which shows plots of median *K*^trans^ (Figure 2B) and median *v*_e_ (Figure 2C) *versus* IFP.

**Figure 3:**
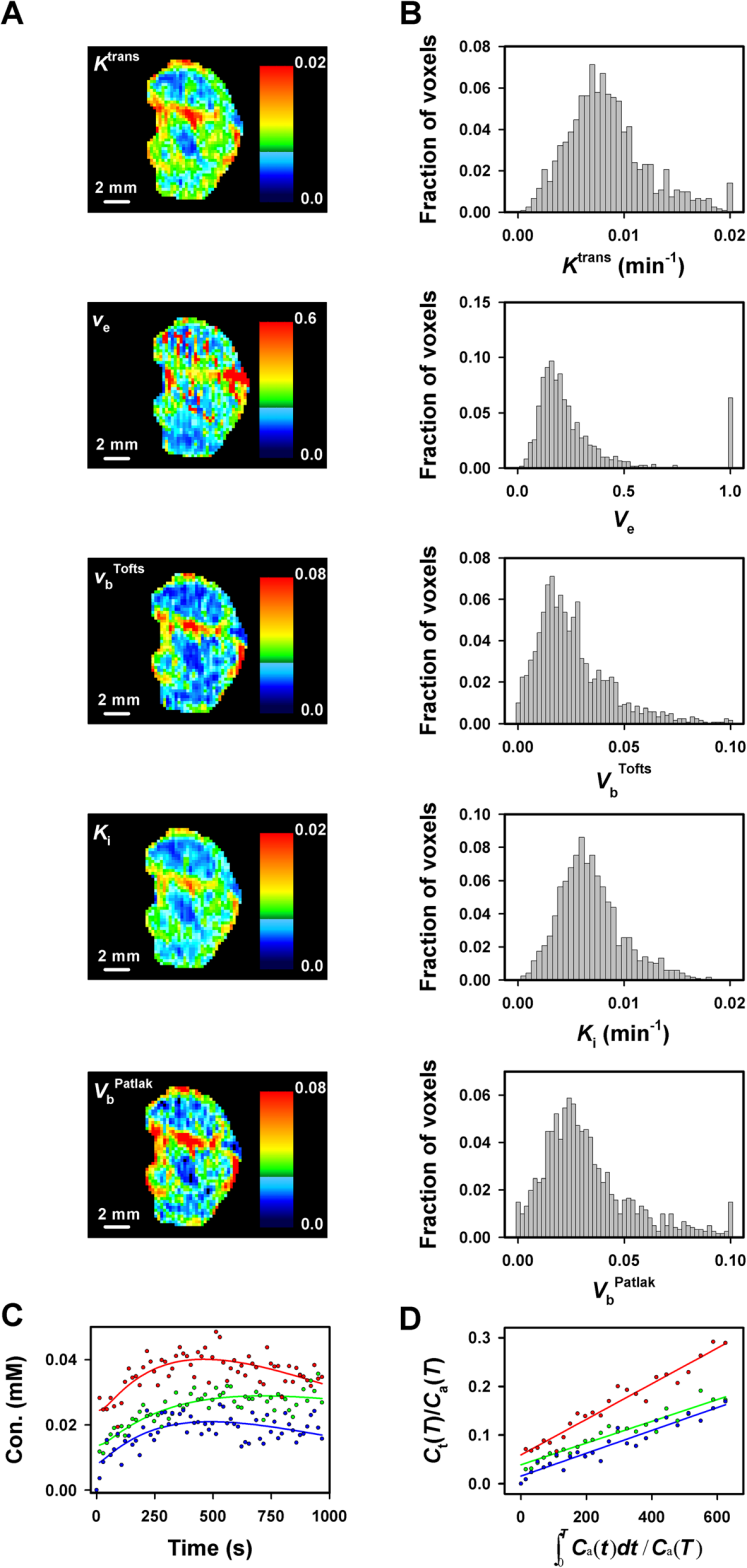
**DCE-MRI data for CK-160 cervical carcinoma xenografts imaged with gadomelitol as contrast agent.** (**A**) The parametric images of *K*^trans^, *v*_e_, *V*_b_^Tofts^, *K*_i_, and *V*_b_^Patlak^ of a representative tumor. (**B**) The *K*^trans^, *v*_e_, *V*_b_^Tofts^, *K*_i_, and *V*_b_^Patlak^ frequency distributions of the same tumor. (**C**) Gadomelitol concentration *versus* time for three representative single voxels of the same tumor. The curves were fitted to the data by using the Tofts pharmacokinetic model. (**D**) *C*_t_(*T*)/*C*_a_(*T*) *versus* ∫*C*_a_(*t*)dt/*C*_a_(*T*) for the same three voxels. The curves were fitted to the data by using the Patlak pharmacokinetic model.

Fifteen tumors were subjected to DCE-MRI with gadomelitol as contrast agent. Parametric images of *K*^trans^, *v*_e_, *V*_b_^Tofts^, *K*_i_, and *V*_b_^Patlak^ and the corresponding *K*^trans^, *v*_e_, *V*_b_^Tofts^, *K*_i_, and *V*_b_^Patlak^ frequency distributions of a representative tumor are presented in Figure 3A and 3B. The tumors were heterogeneous in all parameters. In general, the *K*^trans^ images were similar to the *K*_i_ images and the *V*_b_^Tofts^ images were similar to the *V*_b_^Patlak^ images. Good curve fits were obtained with both pharmacokinetic models. Examples are presented in Figure 3, which refers to three representative single voxels and shows the experimental data and the best curve fits obtained with the Tofts model (Figure 3C) and the Patlak model (Figure 3D).

**Figure 4:**
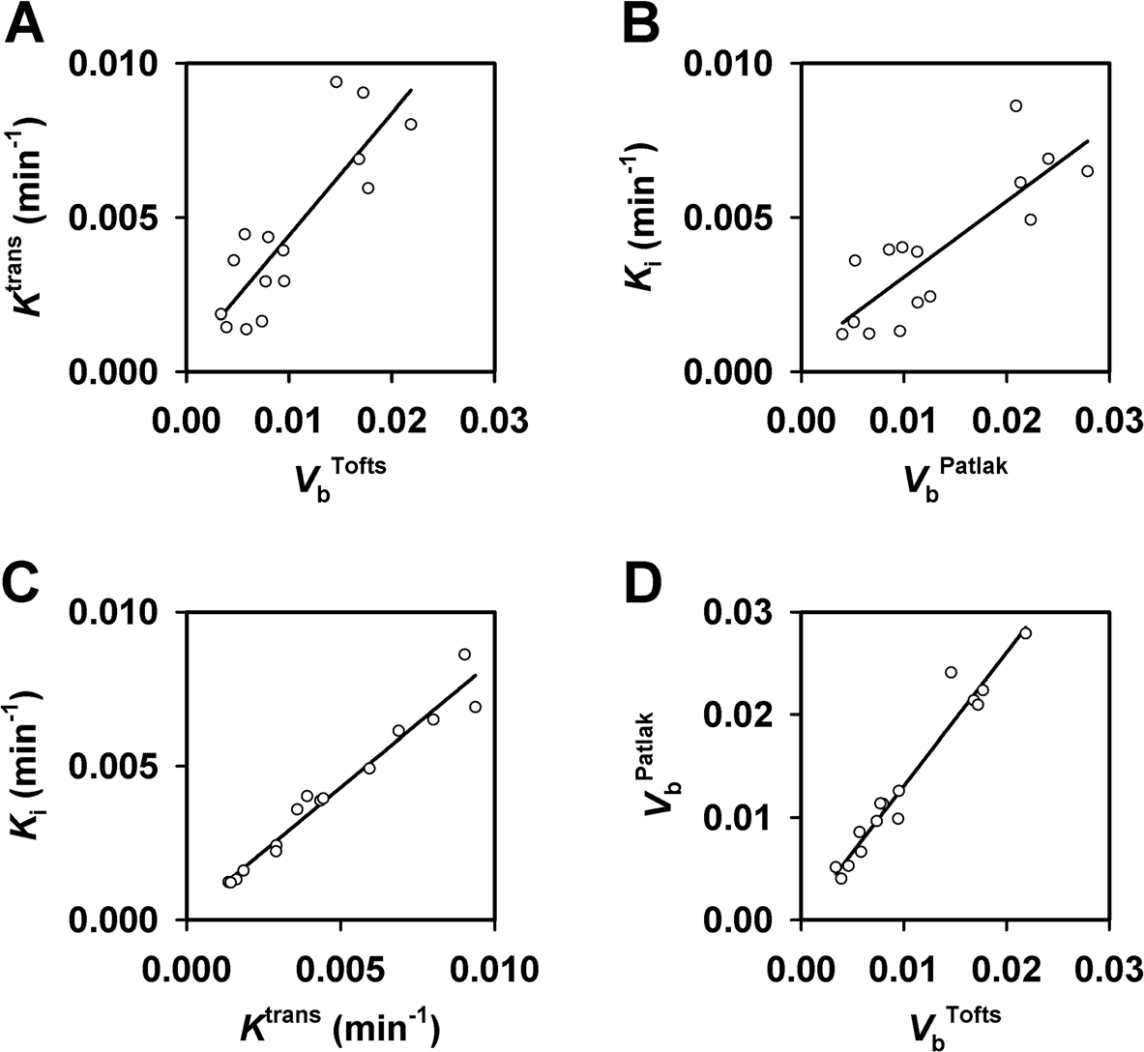
**DCE-MRI data for CK-160 cervical carcinoma xenografts imaged with gadomelitol as contrast agent.** (**A**) Median *K*^trans^*versus* median *V*_b_^Tofts^. (**B**) Median *K*_i_*versus* median *V*_b_^Patlak^. (**C**) Median *K*_i_*versus* median *K*^trans^. (**D**) Median *V*_b_^Patlak^*versus* median *V*_b_^Tofts^. The points represent single tumors. The curves were fitted to the data by linear regression analysis.

As indicated by the images in Figure 3A, the parameters derived from the pharmacokinetic analyses were correlated with each other. This is illustrated in Figure 4, which shows plots of median *K*^trans^*versus* median *V*_b_^Tofts^ (Figure 4A; *P* < 0.0001 and *R*^2^ = 0.72), median *K*_i_*versus* median *V*_b_^Patlak^ (Figure 4B; *P* = 0.0001 and *R*^2^ = 0.69), median *K*_i_*versus* median *K*^trans^ (Figure 4C; *P* < 0.0001 and *R*^2^ = 0.96), and median *V*_b_^Patlak^*versus* median *V*_b_^Tofts^ (Figure 4D; *P* < 0.0001 and *R*^2^ = 0.95).

**Figure 5:**
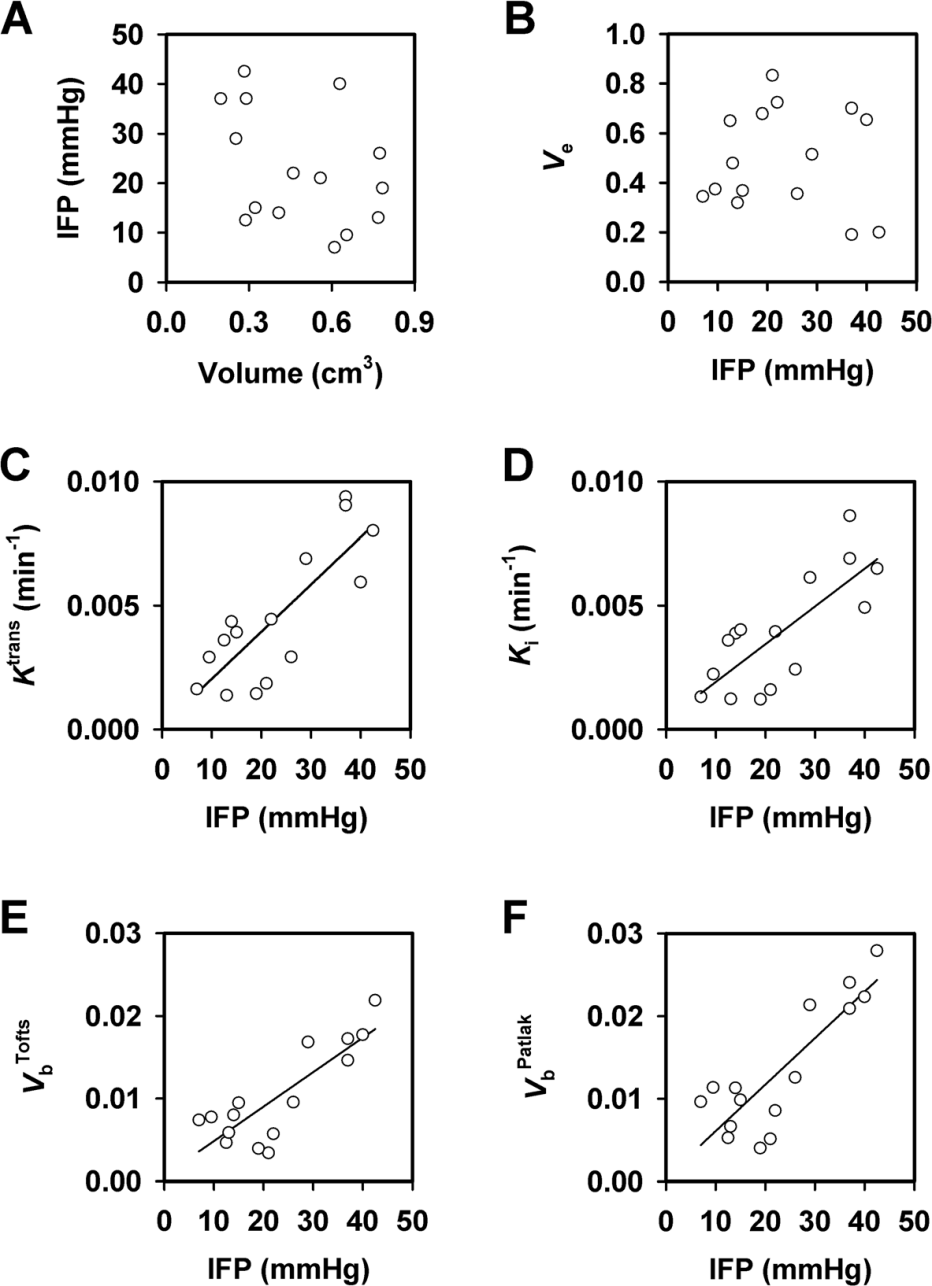
**DCE-MRI and IFP data for CK-160 cervical carcinoma xenografts imaged with gadomelitol as contrast agent.** (**A**) IFP *versus* tumor volume. (**B**) Median *v*_e_*versus* IFP. (**C**) Median *K*^trans^*versus* IFP. (**D**) Median *K*_i_*versus* IFP. (**E**) Median *V*_b_^Tofts^*versus* IFP. (**F**) Median *V*_b_^Patlak^*versus* IFP. The points represent single tumors. The curves were fitted to the data by linear regression analysis.

Tumor IFP was measured immediately after the DCE-MRI also in this experiment and, again, there was no correlation between IFP and tumor volume (Figure 5A). Moreover, there was no correlation between median *v*_e_ and IFP (Figure 5B). However, significant positive correlations were found between median *K*^trans^ and IFP (Figure 5C; *P* = 0.0002 and *R*^2^ = 0.66), median *K*_i_ and IFP (Figure 5D; *P* = 0.0008 and *R*^2^ = 0.59), median *V*_b_^Tofts^ and IFP (Figure 5E; *P* = 0.0001 and *R*^2^ = 0.70), and median *V*_b_^Patlak^ and IFP (Figure 5F; *P* < 0.0001 and *R*^2^ = 0.72).

## Discussion

Cervical cancer patients with primary tumors with high IFP have a poor prognosis and may benefit from aggressive treatment, implying that a noninvasive method for assessing IFP in cervical carcinomas is needed [8-10]. The potential usefulness of DCE-MRI with Gd-DTPA or gadomelitol as contrast agent was evaluated in this preclinical study. Significant correlations between DCE-MRI-derived parameters and IFP were found for gadomelitol, whereas significant correlations could not be detected for Gd-DTPA.

CK-160 human cervical carcinoma xenografts were used as experimental tumor models. This tumor line was established from a pelvic lymph node metastasis of a 65-year-old woman with a well-differentiated (histological grade I) keratinizing primary tumor. The histological appearance of CK-160 xenografts is similar to that of the donor patient’s tumor, and there is evidence that the metastatic pattern and radiation sensitivity of the donor patient’s tumor are retained after xenotransplantation [16]. The physiological microenvironment differs substantially among individual CK-160 xenografts, and the intertumor heterogeneity in several pathophysiological parameters is similar to that reported for cervical carcinomas in humans [16,23]. Thus, IFP values ranging from 6.5 to 45 mmHg were measured in this work, which is comparable to the IFP values of up to ~50 mmHg that have been recorded in untreated tumors in cervical cancer patients [3-5,8-10]. Elevated IFP in tumors is partly a consequence of abnormalities in the microvascular network, and the architecture and function of the microvascular network may differ substantially among individual tumors of the same experimental line as a consequence of stochastic processes influencing tumor angiogenesis shortly after transplantation and during tumor growth. In CK-160 tumors as well as in tumors of several other experimental lines, these stochastic processes result in an intertumor heterogeneity in IFP similar to that observed in tumors in man [1,7,11,16]. Consequently, tumors of the CK-160 cervical carcinoma line should be excellent preclinical models for studying the question addressed in the present work.

The DCE-MRI was carried out at 1.5 T at a spatial resolution of 0.23 × 0.23 × 2.0 mm^3^ and a time resolution of 14 s. By subjecting the same tumors to Gd-DTPA-based DCE-MRI twice, we have shown that our DCE-MRI method produces highly reproducible *K*^trans^ and *v*_e_ images [20]. Moreover, Monte Carlo analysis has revealed that the signal-to-noise ratio is sufficiently high that the *K*^trans^ and *v*_e_ images are not influenced significantly by noise [24], a finding that was confirmed to be valid also in this work. However, our DCE-MRI method has some limitations. Thus, only a single axial slice through the tumor center was scanned, and the influence of any interanimal variation in the arterial input function was ignored. However, as discussed in detail previously, the benefit of considering these factors is small in standardized preclinical studies [17]. The strengths and weaknesses of our DCE-MRI procedure have been reviewed thoroughly elsewhere [17,20,24].

The DCE-MRI series were analyzed with the Tofts iso-directional transport model [14] and the Patlak uni-directional transport model [19]. The main difference between these models is that any transfer of contrast agent from the interstitium to the blood is taken into consideration in the Tofts model whereas the Patlak model assumes irreversible transfer of contrast from the blood to the interstitial space. By neglecting the redistribution rate constant in the Tofts model, the general equation of the Patlak model is obtained with *K*^trans^ = *K*_i_ and *V*_b_^Tofts^ = *V*_b_^Patlak^[13].

The Gd-DTPA data could not be analyzed reliably with the Patlak model because the condition of uni-directional transport was not fulfilled (i.e., the plots of *C*_t_(*T*)/*C*_a_(*T*) *versus* ∫*C*_a_(*t*)dt/*C*_a_(*T*) were not linear). The Tofts model gave good fits to the Gd-DTPA data, but the uptake of Gd-DTPA was too fast relative to the temporal resolution of the DCE-MRI to obtain reliable values for *V*_b_^Tofts^. The analysis of the Gd-DTPA data with the Tofts model was therefore carried out by setting *V*_b_^Tofts^ equal to zero, a simplification that has been shown to have insignificant consequences for the numerical values of *K*^trans^ and *v*_e_ in tumors with blood volume fractions of less than 5% [13]. According to the gadomelitol data in Figure 5, the blood volume fraction is less than 3% in CK-160 tumors. Consequently, it is unlikely that there were correlations between *K*^trans^ and IFP and/or *v*_e_ and IFP that were not detected because of inadequate pharmacokinetic analysis of the Gd-DTPA data.

The gadomelitol data on the other hand could be analyzed reliably with both pharmacokinetic models, and the results did not differ significantly between the models. Thus, the *K*^trans^ images were similar to the *K*_i_ images and the *V*_b_^Tofts^ images were similar to the *V*_b_^Patlak^ images. Furthermore, significant correlations were found between median *K*^trans^ and median *K*_i_ and between median *V*_b_^Tofts^ and median *V*_b_^Patlak^. However, median *K*_i_ was somewhat lower than median *K*^trans^ and median *V*_b_^Patlak^ was somewhat higher than median *V*_b_^Tofts^, probably because the condition of uni-directional transport was not fulfilled completely.

*V*_b_^Tofts^ is assumed to represent tumor blood volume fraction, whereas the physiological interpretation of *K*^trans^ is more complex because *K*^trans^ is influenced by the blood perfusion and the vessel surface area of the imaged tumor and the vessel wall permeability of the contrast agent [14]. In high-permeability situations where the flow of contrast across the vessel wall is limited by the blood supply (i.e., low-molecular-weight contrast agents and leaky, immature blood vessels), *K*^trans^ is determined primarily by the tumor blood perfusion. In low-permeability situations where the flow of contrast across the vessel wall is limited by the vessel wall itself (i.e., high-molecular-weight contrast agents and mature vessels), *K*^trans^ is determined primarily by the permeability surface area product, *PS*, where *P* represents vessel wall permeability and *S* represents vessel surface area per unit tumor volume. CK-160 tumors have mature blood vessels embedded in bands of connective tissue [25], and because the uptake of gadomelitol was slow compared with that of Gd-DTPA, it is likely that the *K*^trans^ of gadomelitol was determined mainly by the permeability surface area product rather than the blood perfusion. Moreover, because strong correlations were found between *K*^trans^ and *V*_b_^Tofts^ and between *K*_i_ and *V*_b_^Patlak^, the differences in *K*^trans^ and *K*_i_ among the individual CK-160 tumors was most likely a consequence of differences in vessel surface area rather than vessel wall permeability.

Significant correlations were found between the *K*^trans^, *K*_i_, *V*_b_^Tofts^, and *V*_b_^Patlak^ of gadomelitol on the one hand and IFP on the other. Although the transcapillary permeability of gadomelitol appears to be low in CK-160 tumors, the hydraulic conductivity of the vessel walls may be high. The differences in IFP among tumors with high vessel wall hydraulic conductivity are mainly a consequence of differences in viscous and geometric resistance to blood flow [1,6]. Several microvascular parameters may cause high resistance to blood flow in tumor tissues, including small vessel diameters, long vessel segment lengths, and high vessel tortuosity [26]. In contrast to small vessel diameters and long vessel segment lengths, high vessel tortuosity may be associated with high vascular fractions in tumors, as shown for U-25 melanoma xenografts [27]. Consequently, the correlations between *K*^trans^ and IFP, *K*_i_ and IFP, *V*_b_^Tofts^ and IFP, and *V*_b_^Patlak^ and IFP in CK-160 tumors most likely appeared because high vessel tortuosity resulted in high IFP as well as high blood volume fractions and large vessel surface areas.

Previously, we have investigated the potential of DCE-MRI as a method for assessing IFP in tumors by using orthotopic A-07 melanoma xenografts as experimental tumor models [21,28]. When Gd-DTPA was used as contrast agent, a significant inverse correlation was found between *K*^trans^ and IFP [28]. With gadomelitol as contrast agent, significant postive correlations were found between *V*_b_^Tofts^ and IFP and between *V*_b_^Patlak^ and IFP [21]. There was no correlation between *K*^trans^ and *V*_b_^Tofts^ or *K*_i_ and *V*_b_^Patlak^ in that study and, hence, no correlation between *K*^trans^ and IFP or *K*_i_ and IFP. The observations reported here for CK-160 cervical carcinomas thus differ substantially from those reported for the A-07 melanomas. The apparent discrepancies are most likely a consequence of differences in the microvascular network and in the quantity and distribution of connective tissue. The fraction of connective tissue is >30% and the fraction of vessels associated with connective tissue is ~80% in CK-160 tumors, whereas in A-07 tumors, the fraction of connective tissue is <10% and the fraction of vessels associated with connective tissue is ~10% [25]. Moreover, the majority of the microvessels in CK-160 cervical carcinomas are surrounded by broad bands of connective tissue, whereas most microvessels in A-07 melanomas are not separated from the parenchyma by connective tissue [25]. In fact, because the transvascular and interstitial transport of MR contrast agents is inhibited by connective tissue and the extent of inhibition is influenced significantly by the molecular weight of the contrast agent, we expected that the results from the present study of CK-160 tumors would differ from those of our previous studies of A-07 tumors, and this expectation was verified to be valid.

Taken together, our studies of A-07 melanoma xenografts and CK-160 cervical carcinoma xenografts suggest that assessment of the IFP of tumors by DCE-MRI may require different strategies for different histological types of cancer, depending on the resistance to transcapillary transport of MR contrast agents. For tumors similar to the A-07 tumors, which show low resistance to transcapillary transport, DCE-MRI parameters related to blood perfusion (e.g., the *K*^trans^ of low-molecular-weight contrast agents like Gd-DTPA) and to blood volume fraction (e.g., the *V*_b_^Tofts^ and *V*_b_^Patlak^ of intermediate-sized contrast agents like gadomelitol) may provide information on tumor IFP. For tumors similar to the CK-160 tumors, which show increased resistance to transcapillary transport, information on tumor IFP may be derived from DCE-MRI parameters related to the permeability surface area product (e.g., the *K*^trans^, *K*_i_, *V*_b_^Tofts^, and *V*_b_^Patlak^ of intermediate-sized contrast agents like gadomelitol).

It should be noticed, however, that these suggestions are based on studies involving only one tumor line with little connective tissue and only one tumor line with substantial quantities of connective tissue. This is a significant limitation, and further studies involving several tumor lines of each category are needed before definite conclusions can be drawn.

It should also be noticed that Haider et al. [12] have investigated whether DCE-MRI with Gd-DTPA as contrast agent may provide information on the IFP of the primary tumor of patients with cervical cancer. They found weak but significant inverse correlations between two *K*^trans^-related parameters (rk_trans_ and IAUC_60m_) and IFP and suggested that rk_trans_ and IAUC_60m_ may be of value in assessing the IFP and, hence, the clinical behavior of cervical carcinomas. These observations were not confirmed in the present study of CK-160 cervical carcinoma xenografts. Our study rather suggests that the *K*^trans^ of Gd-DTPA may not be associated with IFP in cervical carcinomas and, furthermore, that assessment of IFP in cervical carcinomas by DCE-MRI may require contrast agents with higher molecular weights than Gd-DTPA.

## Conclusions

As opposed to Gd-DTPA based DCE-MRI, DCE-MRI with gadomelitol as contrast agent may provide information on the IFP of cervical carcinoma xenografts. Because our study involved tumors of a single line only and only one contrast agent was investigated, further preclinical studies are needed. These studies should include several cervical carcinoma xenograft lines and several contrast agents differing in molecular weight. Furthermore, clinical attempts to develop a DCE-MRI assay of the IFP of cervical carcinomas should involve medium-sized contrast agents like gadomelitol.

## Competing interests

The authors declare that they have no competing interests.

## Authors’ contributions

TH was involved in conceiving the study, designing and performing experiments, analyzing and interpreting data, carrying out statistical analyses, and preparing the manuscript. CE was involved in designing experiments, interpreting data, and preparing the manuscript. EKR was involved in conceiving the study, interpreting data, and preparing the manuscript. All authors read and approved the final manuscript.

## Pre-publication history

The pre-publication history for this paper can be accessed here:

http://www.biomedcentral.com/1471-2407/12/544/prepub
